# First person – Joel Nyarkoh

**DOI:** 10.1242/bio.062947

**Published:** 2026-10-23

## Abstract

First Person is a series of interviews with the first authors of a selection of papers published in Biology Open, helping researchers promote themselves alongside their papers. Joel Nyarkoh is first author on ‘ Rapalink-1 reveals an mTOR- and p53-regulated transcriptional program in Neuro-2a cells’, published in BiO. Joel is a Master's student in the lab of David M. Feliciano at Clemson University, Clemson, SC, USA, investigating the cellular and molecular mechanisms that determine whether neural stem cells promote disease progression or support nervous system recovery.

**Figure:**
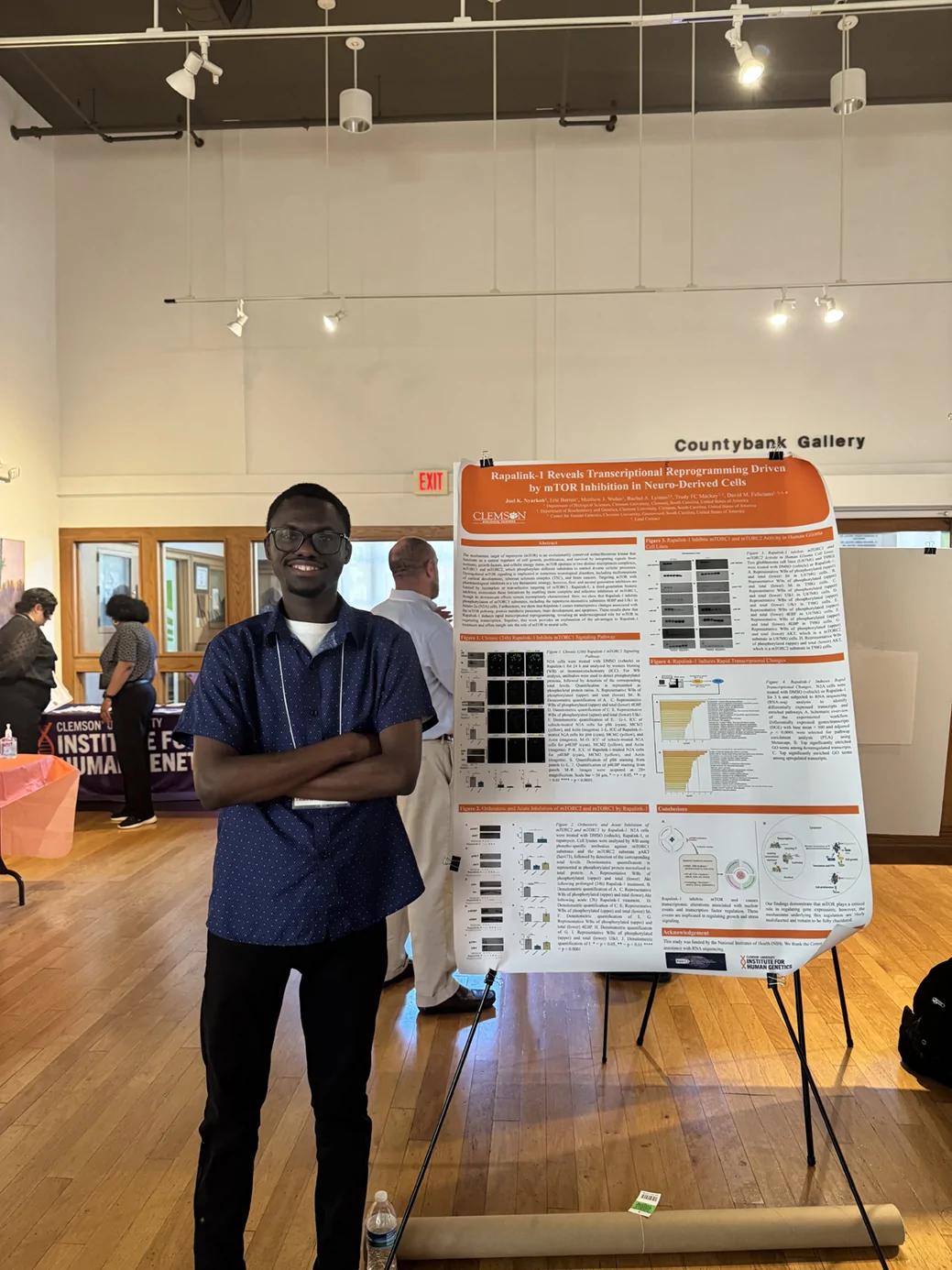
Joel Nyarkoh

**Describe your scientific journey and your current research focus**

My scientific journey began when I was admitted to the Medical Laboratory Science programme at the University of Cape Coast (UCC). Through this programme, I developed a strong foundation in understanding the human body through courses in molecular biology, physiology, anatomy and neuropathology. I completed the programme with an undergraduate capstone project investigating surrogate markers of inflammation using the full blood count dataset for the early detection of malaria-related inflammation. This work led to my first publication and got me hooked on biomedical research. After graduating, I worked as a research and teaching assistant at the UCC Medical School, where I led a systematic review of how neural stem/progenitor-cell therapy helps the brain recover after stroke. This work drew me toward neural stem-cell biology, particularly toward understanding how these cells develop. This interest brought me to Professor David Feliciano's laboratory at Clemson University, roughly 5600 miles from home, where I began studying how the mTOR signalling pathway becomes dysregulated in tuberous sclerosis complex. For my Master's research, I used Rapalink-1, a newer-generation mTOR inhibitor, together with RNA sequencing to investigate how neural cells respond when mTOR signalling is suppressed. My focus has shifted since I started. I began by studying how neural stem cells help repair tissue after injury, and I am now interested in how dysregulated signalling can push neural cells toward pathological states. Ultimately, I want to understand why these cells support repair in some settings but may contribute to abnormal growth in others.

**Who or what inspired you to become a scientist?**

Growing up as a first-generation college student, I developed an early interest in the natural sciences and mathematics. I often performed at the top of my classes in these subjects, and my teachers recognized my potential and encouraged me to pursue a career in science. Motivated by their encouragement, I chose biology, chemistry, physics and mathematics electives in high school. Before completing high school, I had a harrowing experience when my father suffered a devastating stroke. Watching his rapid decline and eventual death made me wonder whether earlier diagnosis or better treatment could have given him a fighting chance. This experience sparked my interest in neurological disease and motivated me to pursue a career in biomedical science, with the hope of contributing to earlier diagnoses and treatments that can prevent similar tragedies. My research mentors have also shaped my perspective on scientific research and taught me how to navigate the rapidly evolving landscape of science. I draw inspiration from the experiences and guidance they share with me as I continue to grow into an independent scientist.

Our study shows that Rapalink-1 strongly suppresses mTOR activity in cell lines derived from central nervous system tumours, including glioblastoma and neuroblastoma

**How would you explain the main finding of your paper?**

Our study shows that Rapalink-1 strongly suppresses mTOR activity in cell lines derived from central nervous system tumours, including glioblastoma and neuroblastoma. mTOR is part of a nutrient-sensitive pathway that helps control cell growth. When this pathway is not properly regulated, it can become overactive and contribute to abnormal cell growth. Earlier mTOR inhibitors have some limitations. First-generation drugs do not completely block all mTORC1 activity, while second-generation drugs can affect both mTORC1 and mTORC2 without sufficient selectivity. We confirmed that Rapalink-1 strongly reduced the phosphorylation of several mTOR targets in neural-derived cells (glioblastoma cell lines and Neuro-2a cells), including ULK1, which is involved in autophagy, a process cells use to recycle their components. We then used RNA sequencing to examine how a short treatment with Rapalink-1 changed gene expression in Neuro-2a cells. The treatment changed the expression of genes involved in mTOR signalling, cell growth, brain development and cell survival. Many of these associated transcripts were linked to p53, a protein that helps cells respond to stress, and Rapalink-1 also changed p53 levels.

**What are the potential implications of this finding for your field of research?**

Overall, our findings suggest that Rapalink-1 affects neural cells through both mTOR- and p53-associated programmes, giving us a better understanding of how the drug works and what may influence its therapeutic potential. This work may help guide the development of treatments for conditions involving excessive mTOR activity, including tuberous sclerosis complex and some tumours.

**Figure:**
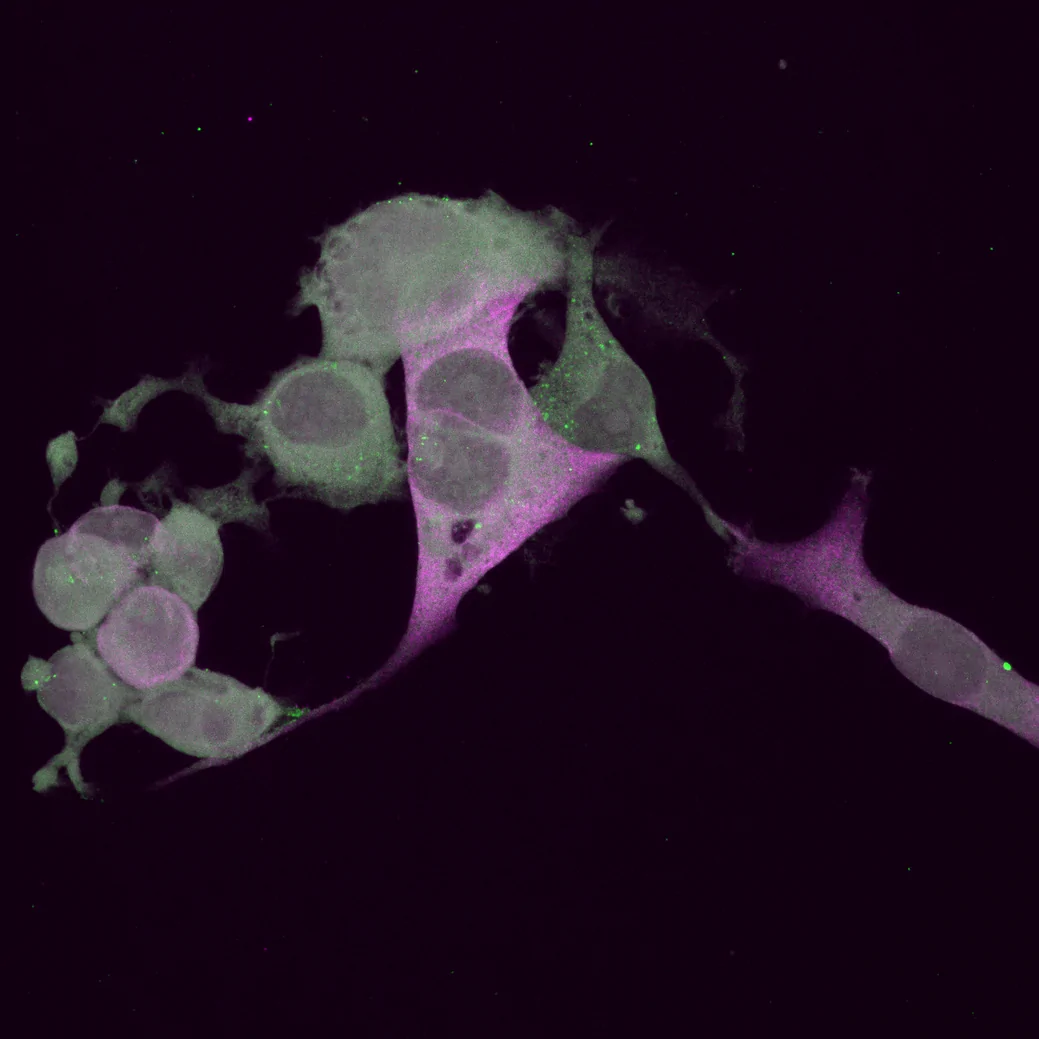
63× confocal microscopy image of Neuro-2a cells stained for Actin (green) and pS6 (magenta).

**Which part of this research project was the most rewarding?**

The most rewarding part of my research was presenting my findings for the first time in the Advances in Human Genetics seminar series of the Institute for Human Genetics. These meetings brought together scientists from biology, genetics, biochemistry and bioengineering at Clemson University. I felt honoured to share my work with such a broad scientific community. What stood out most was the encouragement I received from Professor Robert Anholt, Provost's Distinguished Professor of Genetics and Biochemistry at Clemson University. After hearing about my research, he remarked that I might one day be on the path to winning a Nobel Prize. Although his comment was partly in jest, it was very encouraging, and what meant the most to me was that an established scientist recognized promise in my work and encouraged me to aim high.

**What do you enjoy most about being an early-career researcher?**

I love the iterative nature of research. I enjoy how a hypothesis-driven experiment can answer one question while opening new ones, allowing us to build on existing knowledge and expand the field. As an early-career researcher, I find it especially rewarding to be part of this ongoing process of discovery.

**What piece of advice would you give to the next generation of researchers?**

There is nothing more rewarding than seeing your scientific contributions improve people's quality of life. Keep pushing your research forward, and when necessary, do not hesitate to change your strategy or troubleshoot a problem. Even experiments that do not work as expected can bring you closer to a discovery. You may be only one experiment away from finding something important.

**What's next for you?**

After completing my Master's degree, I hope to pursue a PhD focused on understanding the cellular and molecular mechanisms that determine whether neural stem cells contribute to disease progression or support recovery of the nervous system. I also hope to expand my experience from cell-based models to more complex systems, including brain organoids and animal models of neurological disease.
